# Guiding immunotherapy based on the oxford classification activity score in IgA nephropathy

**DOI:** 10.3389/fendo.2026.1873181

**Published:** 2026-06-23

**Authors:** Jingjing Nong, Yuhong Tang, Jiazhen Yang, Wenli Wei, Licong Su, Sheng Nie, Jun Ou

**Affiliations:** 1 Division of Nephrology, The First Affiliated Hospital of Guilin Medical University, Guilin, Guangxi, China; 2 Division of Nephrology, Nanfang Hospital, Southern Medical University, National Clinical Research Center for Kidney Disease, State Key Laboratory of Organ Failure Research, Guangdong Provincial Clinical Research Center for Kidney Disease, Guangzhou, China

**Keywords:** IgA nephropathy, immunosuppression therapy, Oxford classification, prognosis, supportive care

## Abstract

**Background:**

The efficacy of Oxford classification in guiding immunotherapy management strategies remains a subject of debate. Our study aimed to explore different combinations of Oxford Classification activity scores in guiding immunotherapy for IgA nephropathy.

**Objective:**

We introduced a sum scores to analyze composite pathological score association with immunotherapy for proteinuria remission, aiming to further elucidate the clinical utility of renal histology in immunotherapy decisions.

**Methods:**

A total of 3,196 patients were diagnosed with IgA nephropathy, as confirmed by renal biopsy, between May 2018 and November 2023. However, only 2,389 patients were enrolled after the exclusion criteria were applied. The cohort included 1,211 patients receiving immunosuppressive therapy and 1,178 patients managed with supportive care. Patients with complete outcomes were selected, and the characteristics between groups were balanced using propensity score matching. We subsequently employed both Kaplan-Meier and Cox regression analyses to investigate the impact of immunotherapy on proteinuria remission. We defined SumMEC (the sum of the M, E, and C scores) as a potential immunotherapy responsiveness score. Furthermore, we conducted a subgroup analysis to explore the association between different SumMEC subgroups and the effect of immunotherapy.

**Results:**

A multivariate Cox regression analysis revealed that immunotherapy was associated with a 22% increase in proteinuria remission at 12 months in patients with IgAN (HR 1.22; 95% CI 1.07-1.40; p=0.004). Subgroup analyses demonstrated this benefit only in patients with high SumMEC scores. The effects of immunotherapy and the SumMEC score on 6-month proteinuria remission are comparable to those observed at 12 months.

**Conclusions:**

The use of immunosuppressive agents may improve proteinuria remission in patients with IgA nephropathy. Patients with high SumMEC scores responded positively to immunotherapy.

## 1 Introduction

IgA nephropathy is the most prevalent primary glomerulonephritis worldwide; it was first identified by Berger J. et al. (1) and accounts for 54% of glomerulonephritis cases (2). Additionally, the “four-hit” hypothesis of autoimmune inflammatory activation is thought to play a major role in its pathogenesis (3). The treatment of IgA nephropathy includes general supportive care and immunotherapy, which usually depend on disease activity, including levels of proteinuria and hematuria, renal function, and the patient’s histological features.

The Oxford classification of IgA Nephropathy was initially proposed in 2009 by the International IgA Nephropathy Network and the Renal Pathology Society. It comprises four features: mesangial hypercellularity (M), endocapillary hypercellularity (E), segmental sclerosis (S), and tubulointerstitial fibrosis (T) (4). Since its introduction, sever studies have demonstrated the usefulness of the Oxford classification system as a prognostic tool for IgA nephropathy (5–7). Nevertheless, some controversy remains regarding its potential utility as an indicator for immunotherapy. Numerous studies have demonstrated that the predictive value of the M1 and E1 scores is diminished following immunotherapy (5, 8–10). Relevant repeated renal biopsy studies indicated a reduction in renal pathological changes and clinical presentation following immunotherapy (11). Patients with M1 and E1 scores experience better long-term outcomes and greater proteinuria reduction with steroids than supportive therapy, suggesting these scores may indicate active lesions that respond to steroid treatment (12, 13). Crescents were incorporated into the classification in 2016, amending it to the Oxford classification MEST-C score (14). Studies confirmed that crescents can predict disease prognosis and improve renal outcomes with immunotherapy. These findings suggest that the crescent may also indicate responsiveness to immunotherapy (13, 15–18). However, the STOP-IgA Nephropathy Study did not observe the benefit from immunosuppressive therapy, though histopathological features were not considered (19). Although the TESTING study demonstrated benefits from immunotherapy, it did not mention M or C lesions specifically. In that study, a subgroup analysis revealed a similar trend toward immunotherapy benefit in both E0 and E1 subgroups (20). Other related studies did not report immunotherapeutic responses in patients with E and C lesions (21, 22).Consequently, the current research on the Oxford classification system and immunotherapy is inadequate for guiding clinical therapeutic decisions.

The 2025 KDIGO guidelines state that the International IgAN Prediction Tool and Oxford classification should not guide specific treatment options. Their role is primarily prognostic. Proteinuria is the only validated early biomarker for guiding treatment, with persistent levels ≥0.5 g/L indicating progressive renal decline risk regardless of treatment (23). The severity of pathohistological lesions is correlated with elevated proteinuria levels at the time of biopsy (4, 5). Some studies showed that immunotherapy improved proteinuria remission (24, 25). Furthermore, rapid and sustained proteinuria remission significantly improved renal prognosis (26, 27).

Extensive prior research indicates that the M, E, and C pathological subtypes may represent active pathological manifestations responsive to immunotherapy in patients with IgA nephropathy. However, findings from these studies remain controversial. Most studies have examined individual pathological lesions in relation to renal outcomes or immunotherapy, with few investigating composite Oxford classification scores and immunotherapy benefits for proteinuria remission or renal outcomes. Consequently, evidence-based medical data supporting immunotherapy regimen selection based solely on a single pathological finding is insufficient. Mesangial hypercellularity (M), endocapillary hypercellularity (E), and crescent formations (C) represent three distinct yet complementary pathological processes (14). These lesions may collectively reflect the severity of active immune-mediated glomerular injury (28–30). The Oxford MEST-C scoring system classifies these lesions as categorical variables without weighting their relative contributions to clinical outcomes. Although individual lesions are clinically significant, their combined assessment may provide a more comprehensive reflection of overall disease activity than evaluating any single lesion. Whether immunotherapy response stems from specific lesions or the cumulative effect of concurrent active lesions within the glomeruli remains controversial.

Therefore, we introduced SumMEC (sum of M, E, and C scores) to analyze composite pathological score association with immunotherapy for proteinuria remission, aiming to further elucidate the clinical utility of renal histology in immunotherapy decisions.

## 2 Materials and methods

### 2.1 Study design and population

**Figure 1:**
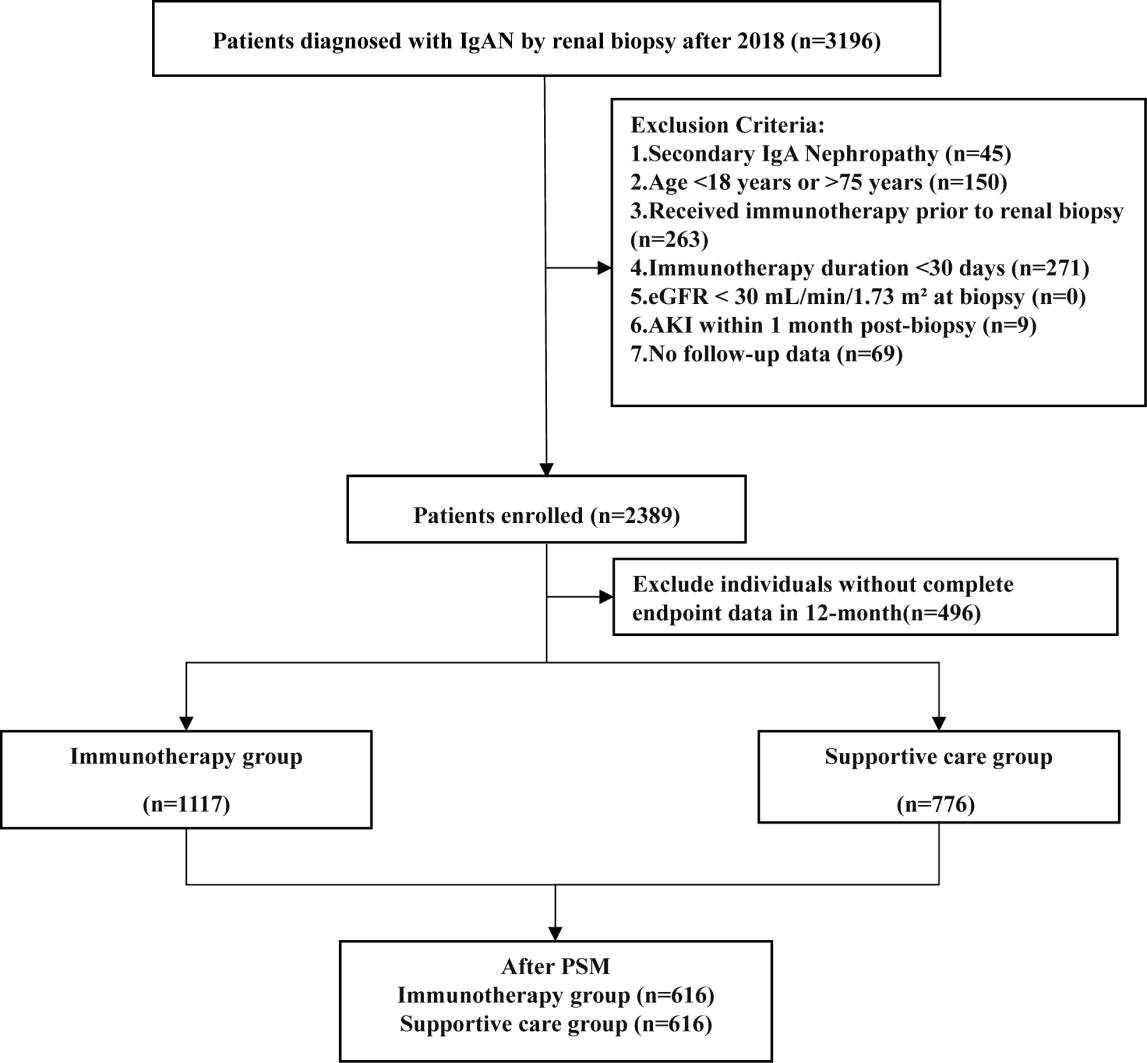
Flow chart of the study.

This retrospective cohort study obtained relevant data from the electronic medical records of the China Renal Data System. All patients with IgA nephropathy included in this study underwent a renal biopsy to confirm their diagnosis. Treatment (supportive care and/or immunosuppression) was initiated within 30 days of the biopsy, regardless of the duration or dose. The non-immunosuppressive supportive therapies used in this study included renin-angiotensin-aldosterone system (RAAS) inhibitors, antihypertensive drugs, and sodium-glucose cotransporter 2 (SGLT2) inhibitors. Immunosuppressive therapies included glucocorticoids, mycophenolate mofetil, cyclophosphamide, cyclosporine, and tacrolimus. The exclusion criteria for this study were as follows: (1) age under 18 or over 75 years; (2) secondary IgAN (including that caused by hepatitis, cirrhosis, IgA vasculitis, or autoimmune diseases); (3) receipt of immunotherapy prior to the renal biopsy; (4) receipt of immunotherapy for less than 30 days; (5) acute kidney injury (AKI) within one month of the biopsy; (6) an estimated glomerular filtration rate (eGFR) of less than 30 ml/min/1.73 m² at the time of biopsy; and (7) a lack of follow-up data (**Figure 1**).

### 2.2 Definitions

Based on International Classification of Diseases-10 (ICD-10) codes, comorbidities, including hypertension and diabetes, were identified at the time of the biopsy. Baseline laboratory indices, such as proteinuria, serum albumin, and serum creatinine, were defined as the earliest values recorded within the 30 days preceding or following the biopsy. Baseline clinical indices, such as blood pressure, were defined as the values recorded within the 30 days following the biopsy. In this study, the eGFR was calculated using the Chronic Kidney Disease Epidemiology Collaboration (CKD-EPI) creatinine equation. Urinary protein remission was defined as a urine albumin-to-creatinine ratio (ACR) of <30 mg/g, a urine total protein-to-creatinine ratio (PCR) of <30 mg/g, negative proteinuria, or a 24-hour urine protein concentration of ≤1 g/L.

Definitions of the Oxford classification: M1, mesangial hypercellularity of ≥4 cells in 1 or more mesangial area present in >50% of glomeruli; E1, presence of endocapillary hypercellularity; S1, presence of segmental glomerulosclerosis and/or adhesion; T0–T2, percentage of cortex showing tubular atrophy and interstitial fibrosis: T0 (≤25% of cortex), T1 (26%–50%), and T2 (>50%); C0–C2, proportion of glomeruli showing cellular and/or fibrocellular crescents: C0 (0%), C1 (1%–24%), and C2 (≥25%). There were no collapsing lesions.

Definition of SumMEC: We defined the SumMEC (the sum of the Oxford classification M, E, and C scores) as a score indicating the likelihood of a response to immunotherapy.

Calculation of SumMEC: In calculating SumMEC, the presence of an M1 pathological lesion was assigned a score of 1 (the same applies to E1 or C1 lesions), while the presence of a C2 lesion was assigned a score of 2.

The SumMEC was evaluated on a 0–4 point scale, where scores of 0–1 indicated a low SumMEC value (e.g., M1E0S0T0C0), and scores of 2–4 indicated a high SumMEC value (e.g., M1E1S1T1C0).

### 2.3 Outcomes

The primary outcome was proteinuria remission at 12 months, and the secondary outcome was proteinuria remission at 6 months. These timepoints were chosen in accordance with the 2025 KDIGO guidelines, which designate proteinuria as the key early biomarker for treatment response in glomerulonephritis, and reflect standard assessment intervals in prior IgA nephropathy studies.

### 2.4 Ethical approval

The study was conducted in accordance with the Declaration of Helsinki and approved by the local Ethics Committee. The ethics approval number for this study is NFEC-2023-409. The committee also approved the informed consent process for the patients.

### 2.5 Statistical analysis

In this study, four variables -BMI, urinary erythrocytes, 24-hour urinary protein, and blood pressure-had high rates of missing values (26%, 14%, 14%, and 12%, respectively). We assumed that these values were missing at random and performed multiple imputation on the missing data using the ‘mice’ package in R software. The normality test for the variables was conducted using the Shapiro-Wilk test. Normally distributed data for continuous baseline variables are presented as the mean ± standard deviation (SD), whereas skewed data are presented as the median and interquartile range (IQR). Categorical variables are reported as numbers (percentages). Quantitative results were compared using unpaired t-tests (for normally distributed data) or Mann-Whitney U tests (for skewed data). Chi-square tests were used to compare categorical variables, such as sex and the Oxford classification. A propensity score was calculated using multivariate logistic regression to generate a 1:1 matched cohort of immunotherapy versus supportive care patients. The model incorporated the following covariates: demographic characteristics (age and sex), laboratory parameters (lipids, serum creatinine, serum albumin, 24-hour urinary protein excretion, and eGFR), comorbidities (hypertension and diabetes), medication history (RAAS inhibitors), and histopathological characteristics (MEST-C classification). A 1:1 nearest-neighbor propensity score matching was performed using the MatchIt package in R, with a caliper width of 0.02 standard deviations. Post-matching covariate balance was assessed using standardized mean differences (SMD), with values of less than 0.1 indicating adequate balance.

Considering that pathological diagnosis, determination of pathological type, and formulation of an individualized immunosuppressive treatment regimen require approximately 1 month after renal biopsy, the study designated 6 months and 12 months post-treatment as observation endpoints, corresponding to 7 months and 13 months post-renal biopsy, respectively. We used Kaplan-Meier and Cox regression analyses to evaluate the effect of immunotherapy on the proteinuria remission rate. However, due to the absence of urine protein results for some study participants, we selected only the patients with IgAN who had complete endpoint data from the pool of 2,389 patients for the 1:1 propensity score matching analysis. We constructed univariate and multivariate Cox regression models based on these data. The multivariate model was adjusted for age, sex, eGFR, proteinuria, SumMEC score, and Oxford classification S and T scores. Additionally, to explore the impact of the SumMEC score on proteinuria remission under immunotherapy, we conducted a subgroup analysis stratified by baseline age (<36 years or ≥36 years), sex, proteinuria level (<1 g/24 h or ≥1 g/24 h), eGFR (<90 ml/min/1.73 m² or ≥90 ml/min/1.73 m²), SumMEC score (<2 points or ≥2 points), and Oxford classification S score (S0 or S1) and T score (T0 or T1 + 2).

All statistical tests were two-sided, and statistical significance was defined as p < 0.05. The results are presented with 95% CIs. All analyses were performed using R software.

## 3 Results

### 3.1 Study population characteristics

In this study, 3,196 patients with IgAN were initially diagnosed via renal biopsy. After the exclusion criteria were applied, 2,389 patients were ultimately enrolled: 1,211 received immunotherapy and 1,178 received supportive care. A total of 1,232 patients were included in the 12-month proteinuria remission outcome cohort following propensity score matching. Among these patients, 616 received immunotherapy and 616 received supportive care (**Figure 1**). **Table 1** presents the baseline characteristics of the cohort, divided by treatment regimen, both before and after matching. The characteristics of the 6-month proteinuria remission population are presented in the supplementary material (**Supplementary Table 1**).

**Table 1:** The baseline characteristics of patients with IgA nephropathy stratified by treatment regimens before and after propensity score matching.

Variables	Before PSM		After PSM	
	Supportive care group (n = 776)	Immunosuppression group (n = 1117)	Supportive care group (n = 616)	Immunosuppression group (n = 616)
Age,yr	36.50 (29.00, 46.00)	33.00 (27.00, 42.00)	36.00 (28.00, 45.00)	35.00 (29.00, 45.00)
Female,n(%)	412 (53.1)	610 (54.6)	334 (54.2)	325 (52.8)
BMI,kg/m2	23.10 (20.75, 25.65)	22.82 (20.52, 25.26)	22.81 (20.54, 25.39)	22.90 (20.57, 25.65)
SBP,mmHg	125.00 (113.00, 135.25)	120.00 (111.00, 130.00)	124.00 (112.00, 134.00)	121.00 (112.00, 130.00)
DBP,mmHg	79.00 (72.00, 88.00)	78.00 (70.00, 86.00)	79.00 (72.00, 87.00)	79.00 (71.00, 86.00)
MAP,mmHg	95.00 (86.67, 103.33)	92.33 (85.00, 100.33)	93.67 (86.25, 102.08)	93.00 (86.00, 100.08)
BUN,mmol/L	5.23 (4.30, 6.66)	5.40 (4.40, 6.93)	5.20 (4.30, 6.65)	5.40 (4.38, 7.00)
Serum creatinine,µmol/L	83.50 (66.00, 108.00)	89.00 (68.00, 115.00)	84.00 (67.00, 109.00)	88.00 (67.38, 116.25)
URBC,counts/HPF	68.40 (22.82, 191.50)	99.90 (33.30, 255.80)	71.68 (25.64, 197.20)	85.00 (28.53, 211.67)
UA,µmol/L	383.00 (320.00, 455.33)	381.00 (313.00, 455.00)	385.00 (317.75, 454.35)	380.50 (312.00, 450.00)
Proteinuria,g/24h	0.83 (0.41, 1.68)	1.19 (0.66, 2.21)	0.87 (0.44, 1.72)	1.08 (0.62, 2.06)
eGFR,ml/min/1.73 m2	101.90 (89.52, 113.01)	101.84 (89.29, 112.79)	101.91 (89.82, 112.58)	101.16 (87.16, 112.79)
TCHO,mmol/L	4.72 (4.05, 5.54)	4.80 (4.14, 5.63)	4.73 (4.08, 5.55)	4.76 (4.07, 5.58)
TG,mmol/L	1.41 (1.04, 2.06)	1.45 (1.03, 2.23)	1.40 (1.03, 2.02)	1.47 (1.04, 2.28)
Serum albumin, g/L	40.10 (36.08, 43.50)	39.00 (35.20, 42.30)	39.90 (35.90, 43.23)	39.60 (35.80, 42.60)
Hb,g/L	130.00 (117.00, 145.00)	127.00 (114.00, 142.00)	130.00 (117.00, 143.00)	127.00 (114.00, 142.00)
Oxford classification, n(%)				
M				
M0	233 (30.0)	248 (22.2)	164 (26.6)	162 (26.3)
M1	543 (70.0)	869 (77.8)	452 (73.4)	454 (73.7)
E				
E0	572 (73.7)	762 (68.2)	449 (72.9)	443 (71.9)
E1	204 (26.3)	355 (31.8)	167 (27.1)	173 (28.1)
S				
S0	194 (25.0)	132 (11.8)	106 (17.2)	102 (16.6)
S1	582 (75.0)	985 (88.2)	510 (82.8)	514 (83.4)
T				
T0	574 (74.0)	733 (65.6)	436 (70.8)	426 (69.2)
T1	154 (19.8)	320 (28.6)	142 (23.1)	144 (23.4)
T2	48 (6.2)	64 (5.7)	38 (6.2)	46 (7.5)
C				
C0	518 (66.8)	556 (49.8)	379 (61.5)	378 (61.4)
C1	242 (31.2)	523 (46.8)	222 (36.0)	218 (35.4)
C2	16 (2.1)	38 (3.4)	15 (2.4)	20 (3.2)
Comorbidities, n (%)	290 (37.4)	331 (29.6)	201 (32.6)	216 (35.1)
Hypertension, n (%)	269 (34.7)	321 (28.7)	193 (31.3)	208 (33.8)
Diabetes, n (%)	55 (7.1)	33 (3.0)	20 (3.2)	29 (4.7)
RAAS, n (%)	704 (90.7)	936 (83.8)	557 (90.4)	563 (91.4)
ACEI, n (%)	211 (27.2)	266 (23.8)	169 (27.4)	163 (26.5)
ARB, n (%)	604 (77.8)	841 (75.3)	474 (76.9)	504 (81.8)
β-blockers, n (%)	81 (10.4)	151 (13.5)	69 (11.2)	73 (11.9)
CCB, n (%)	194 (25.0)	275 (24.6)	147 (23.9)	173 (28.1)
Diuretics, n (%)	119 (15.3)	241 (21.6)	93 (15.1)	131 (21.3)
Lipid-lowering drugs,n (%)	268 (34.5)	523 (46.8)	207 (33.6)	306 (49.7)
SGLT2i,n (%)	10 (1.3)	12 (1.1)	6 (1.0)	10 (1.6)

BMI, body mass index; SBP, systolic blood pressure; DBP, diastolic blood pressure; MAP, mean arterial pressure; BUN, Blood urea nitrogen; U-RBC, urinary red blood cell; HPF, high-power field; UA, uric acid; eGFR, estimated glomerular filtration rate; TCHO, total cholesterol; TG, total glycerides; Hb, Hemoglobin; M, mesangial hypercellularity; E, endocapillary hypercellularity; S, segmental sclerosis; T, interstitial fibrosis/tubular atrophy; C, crescent formations; RASS, renin-angiotensin-aldosterone system; ACEI, angiotensin converting enzyme inhibitor; ARB, angiotensin receptor blockers; CCB, calcium channel blocker; PSM, propensity score matching.

Before matching, patients exhibited higher serum creatinine, urinary erythrocyte, and urinary protein levels; lower eGFRs; and more severe pathological changes in the immunosuppression group. After matching, the immunosuppression group and supportive care groups were similar in demographics, clinical variables, histologic classifications, comorbidities.

Before matching, patients in the immunotherapy group exhibited higher serum creatinine, urinary erythrocyte, and urinary protein levels; lower eGFRs; and more severe pathological changes than those in the supportive care group. After matching, the immunotherapy and supportive care groups were similar with respect to their eGFRs, urinary protein levels, demographics, clinical variables, histologic classifications, comorbidities, and shared medications (SMD < 0.1).

### 3.2 Primary outcome

The immunosuppressive agents used in the study are summarized in the supplementary material (**Supplementary Tables 2**, **3**). Glucocorticoids and MMF were the most common agents, administered either alone or in combination.

**Figure 2:**
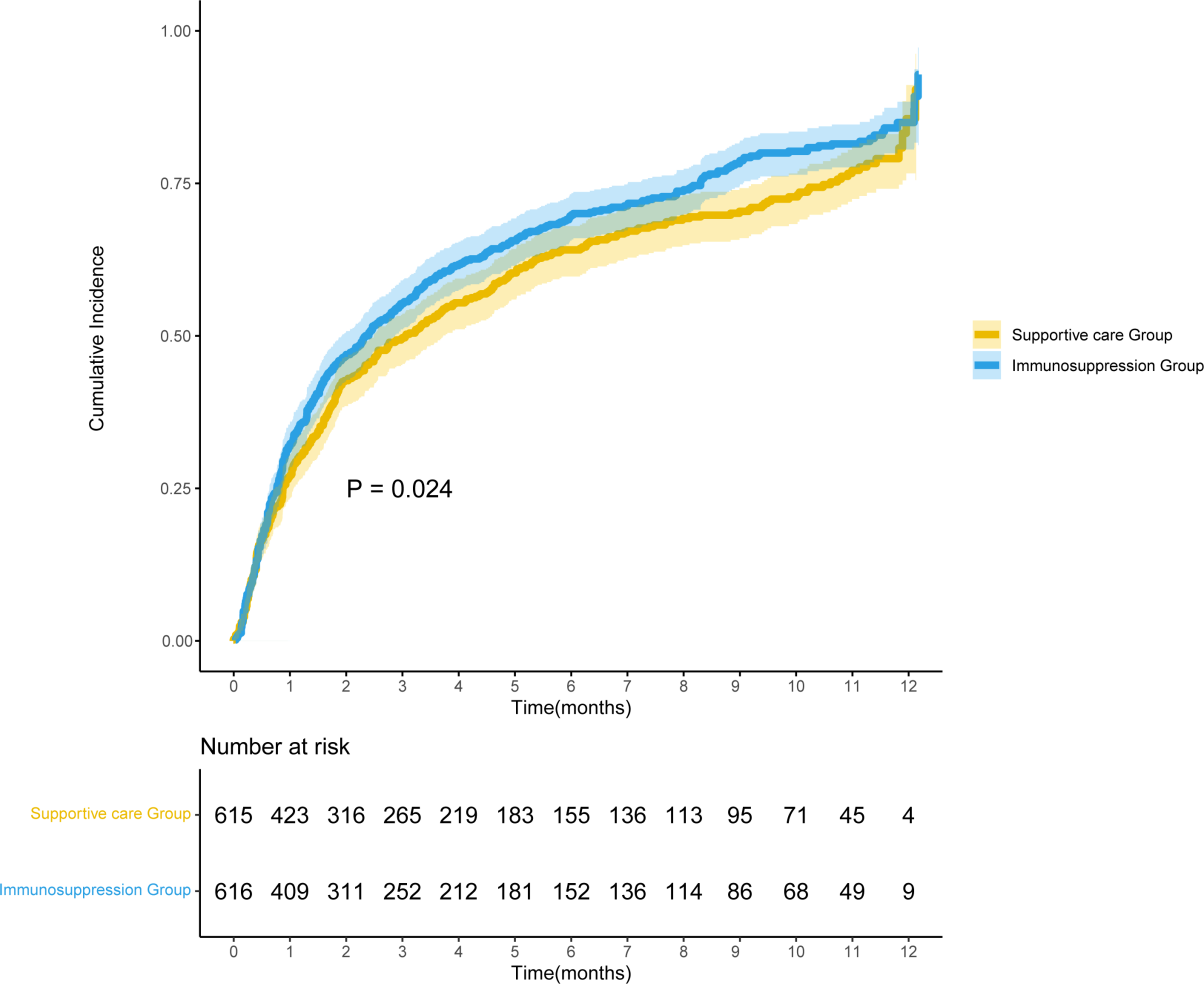
Cumulative incidence of primary outcome in the propensity score-matched cohorts. The proteinuria was reduced among patients who received immunotherapy.

A total of 1,232 patients were included in the propensity score-matched cohort. Kaplan–Meier curves demonstrated a significant decrease in proteinuria among patients who received immunotherapy (HR 1.16; 95% CI 1.02-1.33;p=0.024) (**Figure 2**). During the study period, 875 primary outcome events were observed: 469 events in the immunosuppressive therapy group and 406 events in the supportive care group. A multivariate Cox regression analysis revealed that the immunotherapy group exhibited significant improvement in proteinuria levels at 12 months (adjusted HR 1.22; 95% CI 1.07 -1.40; p=0.004) (**Table 2**).

**Table 2:** The effect of immunosuppressive therapy on primary outcomes in the propensity score-matched (PSM) for all population and SumMEC subgroups.

	Immunosuppression group	Supportive care group		
Outcomes	Events	Events	Crude HR(95%CI)	Adjusted HR(95%CI)
all population	469(76.13%)	406(65.90%)	1.16(1.02-1.33)	1.22(1.07-1.40)
SumMEC≥2	192(72.73%)	144(55.60%)	1.46(1.17-1.81)	1.56(1.24-1.95)

More primary outcome events were observed in the immunosuppressive therapy group. The proteinuria was reduced among patients who received immunotherapy. Furthermore, the effect exhibited more significant in SumMEC subgroup.

**Figure 3:**
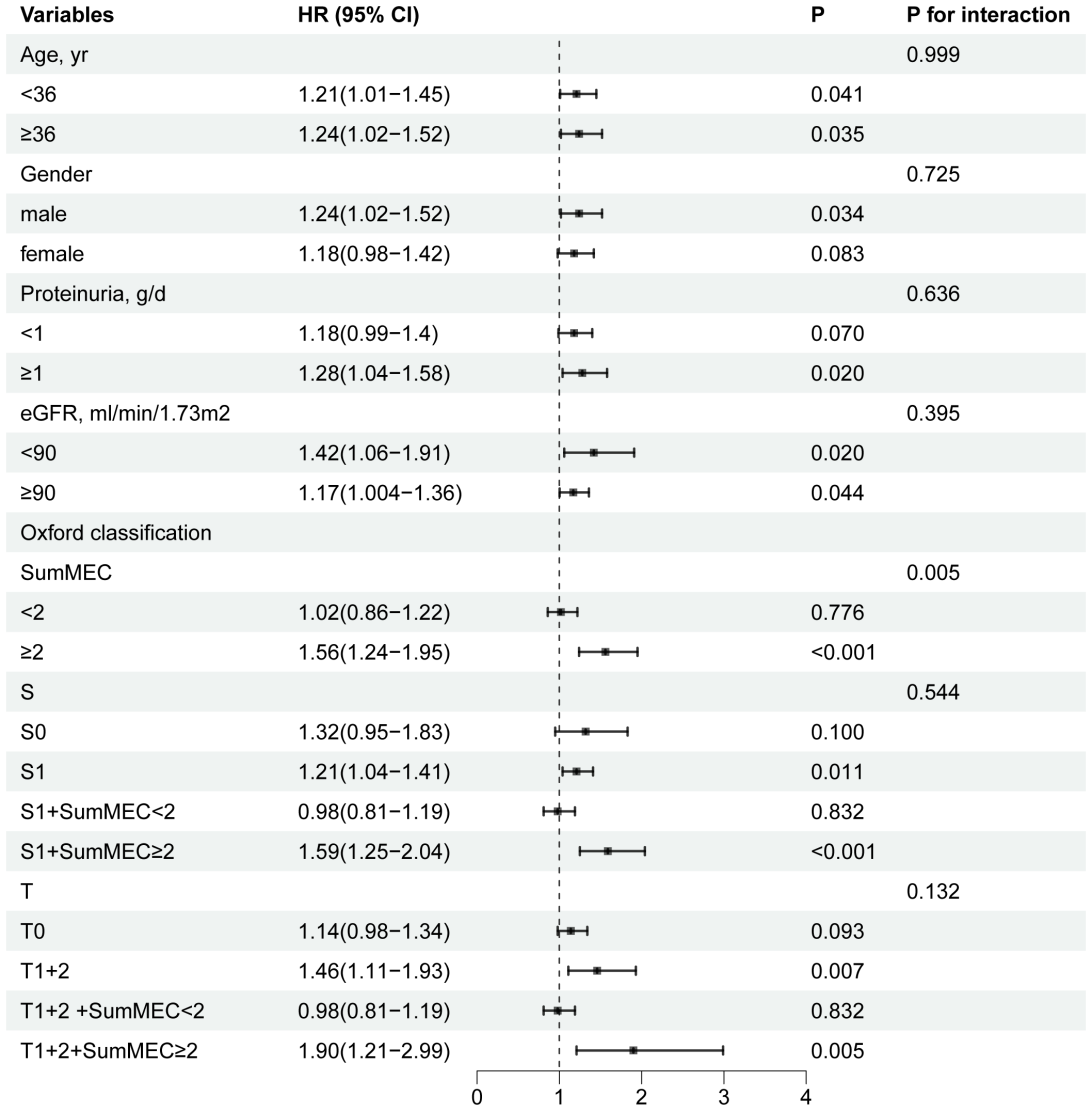
The estimated effects of immunosuppressants on the primary outcome in subgroups Hazard ratio(adjusted for age, sex, eGFR, proteinuria, SumMEC score, S score, and T score). Immunotherapy improved proteinuria remission among patients in the SumMEC ≥ 2 subgroup, as well as the patients in S1 or T1 + 2 subgroup when these patients existed a concomitant SumMEC score ≥ 2.

We performed a subgroup analysis to explore the effect of different SumMEC scores on urinary protein remission following immunotherapy. Among patients in the SumMEC ≥ 2 subgroup, immunotherapy improved proteinuria remission at 12 months (adjusted HR 1.56; 95% CI 1.24–1.95; p for interaction=0.005). Furthermore, patients with S1 scores and SumMEC score ≥ 2 demonstrated proteinuria remission benefits from immunotherapy. The differences between these subgroups were not statistically significant (adjusted HR 1.59; 95% CI 1.25-2.04; p for interaction=0.544). Similarly, immunotherapy improved proteinuria remission in the T1 + 2 score subgroup, but this effect was only present in patients with a concomitant SumMEC score ≥ 2 (adjusted HR 1.90; 95% CI 1.21-2.99; p for interaction=0.132) (**Figure 3**).

### 3.3 Secondary outcome

Additionally, we explored whether immunotherapy had a similar effect on the reduction in the proteinuria concentration at 6 months. A total of 1,180 patients were included in this analysis following propensity score matching.

Kaplan-Meier curves demonstrated a similar effect on reducing proteinuria in patients who received immunotherapy for 6 months (HR 1.15; 95% CI 1.001-1.33; p=0.048) (**Supplementary Figure 1**). A total of 780 outcome events were observed during this study period: 419 events in the immunosuppressive therapy group and 361 events in the supportive care group. A multivariate Cox regression analysis revealed that, compared with supportive care, immunotherapy was associated with a 16% improvement in proteinuria levels (adjusted HR 1.16; 95% CI 1.004-1.33; p=0.043) (**Supplementary Table 4**).

Subgroup analyses indicated that outcomes in the SumMEC ≥ 2 subgroup were similar to those observed at 12 months (adjusted HR 1.54; 95% CI 1.22-1.95; p for interaction=0.005). Additionally, the results for the subgroup of patients with T1 + 2 scores and SumMEC scores ≥ 2 were similar to the 12-month proteinuria remission results (adjusted HR 2.09; 95% CI 1.30-3.36; p for interaction=0.044). Patients with S1 scores and SumMEC scores ≥ 2 also appeared to benefit from proteinuria remission. However, the differences between these subgroups were not statistically significant (adjusted HR 1.55; 95% CI 1.21-2.00; p for interaction=0.657) (**Supplementary Figure 2**).

## 4 Discussion

This study aimed to investigate the association between different combinations of active lesions and the remission of proteinuria to better predict patient responses to immunotherapy.

First, we found that immunotherapy significantly decreased proteinuria. Second, among patients with SumMEC score ≥2, immunotherapy was associated with a more positively improvement in urinary protein remission. Similarly, the S1 or T1 + 2 subgroup experienced beneficial remission, but only among patients with concomitant SumMEC score ≥2. Our results help identify specific IgAN patients through SumMEC subclassification and provide a basis for utilizing the Oxford classification clinically.

IgAN is a mesangial proliferative form of primary glomerulonephritis. It involves an immune-mediated, multistep process that ultimately leads to the deposition of galactose-deficient IgA1 immune complexes in the glomerular mesangium. This deposition promotes mesangial proliferation and triggers a cascade of immune-mediated injury and glomerular inflammation (31, 32). These caused various forms of renal pathological damage which scored by the MEST-C classification. Endocapillary proliferation and Crescent are associated with complement activation and inflammatory response (33, 34). Complement activation may lead to the control of subclinical defects and promote the development of active lesions, such as M1, E1, S1, and crescents. The results of multiple analyses of immunotherapy responses over time, as well as the reduction in pathological changes observed in repeat renal biopsies, suggest that mesangial hypercellularity, endocapillary proliferation, and crescents may represent subtypes that respond well to immunotherapy. Patients with these specific pathologies experience improved renal outcomes with immunotherapy compared to those in supportive care group (11, 17, 35). Active pathological changes driven by inflammatory and immune factors, such as complement, likely underlie the responsiveness of these features to immunotherapy.

Previous studies have reached varying conclusions regarding whether M, E, and C should be considered active reactivity scores. We propose that the discrepancies in previous findings may stem from assessing the histology of only a single lesion type; clinical treatment decisions are rarely based on the histologic findings of an isolated lesion. We hypothesize that a total activity score may better indicate the overall severity of active inflammation. Based on the SumMEC classification, we explore the impact of disease activity and immunotherapy on patient outcomes.

The results of the present study revealed that immunotherapy improved proteinuria remission in the overall population at 12 months (HR 1.16; 95% CI 1.02-1.33), with a similar effect at 6 months (HR 1.15; 95% CI 1.001-1.33). A subgroup analysis revealed that immunotherapy was particularly beneficial for patients with high SumMEC scores. This effect was consistent at both endpoints. A previous Japanese study categorized combinations of the Oxford classification and showed that patients with high reactivity scores experienced improved renal outcomes when treated with immunosuppressants (13). Another study revealed that TRB treatment was linked to decreased proteinuria levels in patients exhibiting active histologic manifestations of mesangial hypercellularity and endocapillary proliferation (12). That subclassification did not include crescent, whereas our study included this lesion in the grading. However, consistent with the results of the Japanese study, we did not observe a benefit of immunotherapy in patients with a low active reactivity score. Although our study endpoint differed from the endpoint in that study, these findings consistently suggest that patients with active disease scores, as defined by our parameters, respond to immunotherapy. Besides, the benefit of proteinuria remission may be more significant in patients with severe active disease who receive immunotherapy. Furthermore, Lv et al. reported that rapid and sustained proteinuria remission improves renal prognosis, emphasizing the importance of achieving proteinuria remission quickly and maintaining it over a long period (26). Overall, our study suggests that immunotherapy is more likely to improve proteinuria remission in patients with high activity scores and that SumMEC scores may be useful for making decisions regarding immunotherapy.

However, our study revealed that the immunotherapy group of patients with S1 and T1 + 2 disease experienced significantly greater improvement in proteinuria levels than the supportive therapy group did, which seems to indicate an immunotherapy benefit. A recent VALIGA cohort study confirmed that immunosuppressive therapy resulted in better outcomes in only two subcategories of S lesions: S1 with NOS and ADH but not PH, and S1 with NOS and PH (36). Another study revealed that patients with CSS lesions benefit from glucocorticoid therapy (18). By contrast, previous studies have consistently shown that T is a chronic lesion, not an immunotherapy responsiveness score (11, 37, 38). Therefore, to further explore the benefits of immunotherapy for patients with S1 or T lesions, we refined the original Oxford classification for the S1 and T1 + 2 subgroups, including the subgroups with or without a high SumMEC score. The results of the study revealed that in patients with either concomitant S1 or T lesions, the ability of immunotherapy to improve proteinuria remission was observed only in patients who also had high SumMEC scores. we hypothesize that these benefits could be due to the coexistence of active lesions.

Our research has several advantages. First, it is a large-scale, multicenter cohort study. Second, we minimized the risk of confounding by balancing the differences between the immunosuppressive treatment group and the supportive care group through propensity score matching. Third, the cohort of patients in this study initiated medication within one month of their renal biopsy, which minimized the impact of pathological findings being influenced by the prior treatment.

This study also has several limitations. First, it was a retrospective observational study, even though we used propensity score matching to equalize baseline feature differences in biopsies, but unknown confounders may still exist. Second, as this study was not a randomized controlled trial (RCT), the follow-up loss may have introduced selection bias affecting the true results. Third, the effectiveness of this study in populations other than the Chinese population needs to be verified. Fourth, this study could not determine the specific subtypes of S1 lesions; thus, whether the immunotherapy benefit observed in S1 patients stems from the aforementioned S subclassification that responds to immunotherapy remains to be explored.

In conclusion, our study demonstrated that immunosuppressive agents may improve urinary protein remission in patients with IgA nephropathy. Patients with high SumMEC scores showed a positive response to immunotherapy.

## Data Availability

The data analyzed in this study is subject to the following licenses/restrictions: The datasets supporting this study cannot be shared publicly to protect participant privacy. Requests to access these datasets should be directed to JN, 3108554434@qq.com.
