# Supplementary material for: Guiding immunotherapy based on the oxford classification activity score in IgA nephropathy
Source: Front Endocrinol (Lausanne). 2026 Jun 23;17:1873181. doi: 10.3389/fendo.2026.1873181 (PMC13337360; doi:10.3389/fendo.2026.1873181)
Supplement: Supplementary file 1 [file DataSheet1.docx]

Supplementary Material

# Supplementary Tables

# Supplementary Table1. Baseline characteristics of patients with IgA nephropathy stratified by treatment regimens before and after propensity score matching .

| **Variables** | **Before PSM** | |  | **After PSM** | |
| --- | --- | --- | --- | --- | --- |
|  | **Supportive care**  **Group (n = 737)** | **Immunosuppression Group (n = 1082)** | | **Supportive care**  **Group (n = 590)** | **Immunosuppression Group (n =590)** |
| Age,yr | 37.00 (29.00, 46.00) | 33.00 (27.00, 42.00) | | 35.00 (28.00, 45.00) | 35.00 (29.00, 45.00) |
| Female,n(%) | 389 (52.8) | 591 (54.6) | | 307 (52.0) | 306 (51.9) |
| BMI,kg/m2 | 23.11 (20.76, 25.68) | 22.77 (20.51, 25.26) | | 22.92 (20.64, 25.39) | 22.82 (20.57, 25.27) |
| SBP,mmHg | 124.00 (113.00, 135.00) | 120.00 (111.00, 130.00) | | 124.00 (113.00, 134.00) | 120.00 (112.00, 130.00) |
| DBP,mmHg | 79.00 (72.00, 87.00) | 78.00 (71.00, 87.00) | | 79.00 (72.00, 87.00) | 78.00 (70.00, 86.00) |
| MAP,mmHg | 94.33 (86.33, 102.67) | 92.67 (85.33, 100.33) | | 93.67 (86.00, 102.33) | 92.50 (84.75, 100.00) |
| BUN,mmol/L | 5.22 (4.30, 6.67) | 5.50 (4.40, 7.00) | | 5.30 (4.25, 6.73) | 5.40 (4.30, 6.94) |
| Serum creatinine,µmol/L | 82.00 (65.00, 108.00) | 89.00 (68.25, 115.00) | | 86.00 (67.00, 109.60) | 87.00 (66.00, 114.75) |
| URBC,counts/HPF | 68.30 (23.10, 189.90) | 101.65 (33.00, 259.58) | | 69.45 (25.76, 188.75) | 81.95 (28.38, 217.20) |
| UA,µmol/L | 383.00 (318.00, 451.00) | 381.00 (312.25, 455.00) | | 385.50 (320.25, 454.85) | 370.00 (305.00, 448.00) |
| Proteinuria,g/24h | 0.82 (0.41, 1.65) | 1.19 (0.66, 2.22) | | 0.94 (0.46, 1.74) | 1.05 (0.56, 1.82) |
| eGFR,ml/min/1.73 m2 | 101.93 (89.83, 113.15) | 101.85 (89.23, 112.63) | | 102.03 (89.73, 112.93) | 101.80 (88.81, 112.73) |
| TCHO,mmol/L | 4.70 (4.05, 5.50) | 4.80 (4.13, 5.62) | | 4.70 (4.07, 5.51) | 4.74 (4.06, 5.50) |
| TG,mmol/L | 1.40 (1.03, 2.05) | 1.46 (1.03, 2.24) | | 1.41 (1.05, 2.04) | 1.50 (1.04, 2.29) |
| Serum albumin, g/L | 40.30 (36.10, 43.60) | 39.10 (35.20, 42.38) | | 39.90 (35.80, 43.20) | 39.80 (36.32, 42.80) |
| Hb,g/L | 130.00 (117.00, 145.00) | 126.50 (114.00, 142.00) | | 130.00 (117.00, 144.00) | 128.00 (116.00, 142.00) |
| Oxford classification, n(%) |  |  | |  |  |
| M |  |  | |  |  |
| M0 | 222 (30.1) | 243 (22.5) | | 155 (26.3) | 158 (26.8) |
| M1 | 515 (69.9) | 839 (77.5) | | 435 (73.7) | 432 (73.2) |
| E |  |  | |  |  |
| E0 | 546 (74.1) | 738 (68.2) | | 429 (72.7) | 430 (72.9) |
| E1 | 191 (25.9) | 344 (31.8) | | 161 (27.3) | 160 (27.1) |
| S |  |  | |  |  |
| S0 | 180 (24.4) | 128 (11.8) | | 106 (18.0) | 105 (17.8) |
| S1 | 557 (75.6) | 954 (88.2) | | 484 (82.0) | 485 (82.2) |
| T |  |  | |  |  |
| T0 | 551 (74.8) | 710 (65.6) | | 428 (72.5) | 418 (70.8) |
| T1 | 140 (19.0) | 310 (28.7) | | 125 (21.2) | 148 (25.1) |
| T2 | 46 ( 6.2) | 62 ( 5.7) | | 37 ( 6.3) | 24 ( 4.1) |
| C |  |  | |  |  |
| C0 | 495 (67.2) | 538 (49.7) | | 368 (62.4) | 363 (61.5) |
| C1 | 226 (30.7) | 506 (46.8) | | 207 (35.1) | 218 (36.9) |
| C2 | 16 ( 2.2) | 38 ( 3.5) | | 15 ( 2.5) | 9 ( 1.5) |
| Comorbidities, n (%) | 272 (36.9) | 321 (29.7) | | 201 (34.1) | 203 (34.4) |
| Hypertension, n (%) | 251 (34.1) | 311 (28.7) | | 192 (32.5) | 195 (33.1) |
| Diabetes, n (%) | 54 ( 7.3) | 32 ( 3.0) | | 26 ( 4.4) | 27 ( 4.6) |
| RAAS, n (%) | 669 (90.8) | 903 (83.5) | | 532 (90.2) | 527 (89.3) |
| ACEI, n (%) | 202 (27.4) | 254 (23.5) | | 163 (27.6) | 159 (26.9) |
| ARB, n (%) | 573 (77.7) | 809 (74.8) | | 455 (77.1) | 464 (78.6) |
| β-blockers, n (%) | 72 ( 9.8) | 143 (13.2) | | 63 (10.7) | 68 (11.5) |
| CCB, n (%) | 180 (24.4) | 263 (24.3) | | 145 (24.6) | 154 (26.1) |
| Diuretics, n (%) | 113 (15.3) | 231 (21.3) | | 89 (15.1) | 108 (18.3) |
| Lipid-lowering drugs,n (%) | 255 (34.6) | 502 (46.4) | | 206 (34.9) | 270 (45.8) |
| SGLT2i,n (%) | 9 ( 1.2) | 12 ( 1.1) | | 6 ( 1.0) | 7 ( 1.2) |

BMI, body mass index; SBP, systolic blood pressure; DBP, diastolic blood pressure; MAP, mean arterial pressure; BUN,Blood urea nitrogen; U-RBC, urinary red blood cell; HPF, high-power field; UA, uric acid; eGFR, estimated glomerular filtration rate; TCHO, total cholesterol; TG, total glycerides; Hb, Hemoglobin; M, mesangial hypercellularity; E, endocapillary hypercellularity; S, segmental sclerosis; T, interstitial fibrosis/tubular atrophy; C,crescent formations; RASS, renin-angiotensin-aldosterone system; ACEI, angiotensin converting enzyme inhibitor; ARB,angiotensin receptor blockers; CCB,calcium channel blocker; PSM,propensity score matching.

Alt Text: Before matching, patients exhibited higher serum creatinine, urinary erythrocyte, and urinary protein levels ; lower eGFRs; and more severe pathological changes in the immunosuppression group. After matching, the immunosuppression group and supportive care groups were similar in demographics, clinical variables, histologic classifications, comorbidities.

**Supplementary Table2. Immunosuppressive therapies for immunotherapy group in primary outcome.**

| **Immunosuppressive therapy** | **n(%)** |
| --- | --- |
| Glucocorticoid | 412（36.9） |
| MMF | 204（18.3） |
| Glucocorticoid+MMF | 364（32.6） |
| Glucocorticoid+TAC | 32（2.9） |
| Glucocorticoid+CsA | 10（0.9） |
| Glucocorticoid+CTX | 53（4.7） |
| other | 42（3.7） |

Abbreviations: MMF, Mycophenolate Mofetil; TAC, Tacrolimus; CsA, Cyclosporine; CTX, cyclophosphamide.

**Supplementary Table3. Immunosuppressive therapies for immunotherapy group in secondery outcome.**

| **Immunosuppressive therapy** | **n(%)** |
| --- | --- |
| Glucocorticoid | 399（36.8） |
| MMF | 199（18.4） |
| Glucocorticoid+MMF | 351（32.4） |
| Glucocorticoid+TAC | 31（2.9） |
| Glucocorticoid+CsA | 10（0.9） |
| Glucocorticoid+CTX | 53（4.9） |
| other | 39（3.6） |

Abbreviations: MMF, Mycophenolate Mofetil; TAC, Tacrolimus; CsA, Cyclosporine; CTX, cyclophosphamide.

**Supplementary Table4. The effect of immunosuppressive therapy on secondary outcomes in the propensity score-matched (PSM) for all population and SumMEC subgroups.**

| Outcomes | **Immunosuppression group** |  | **Supportive care group** | Crude HR(95%CI) | Adjusted HR(95%CI) |
| --- | --- | --- | --- | --- | --- |
|  | events | | events |  |  |
| all population | 419(71.01%) | | 361(61.18%) | 1.15(1.001-1.33) | 1.16(1.004-1.33) |
| SumMEC≥2 | 177(70.51%) | | 122(50.20%) | 1.51(1.20-1.90) | 1.54(1.22-1.95) |

Alt Text: More secondary outcome events were observed in the immunosuppressive therapy group. The proteinuria was reduced among patients who received immunotherapy. Furthermore, the effect exhibited more significant in SumMEC subgroup.

# Supplementary Figures

**Supplementary Figure 1. Cumulative incidence of secondary outcome .**


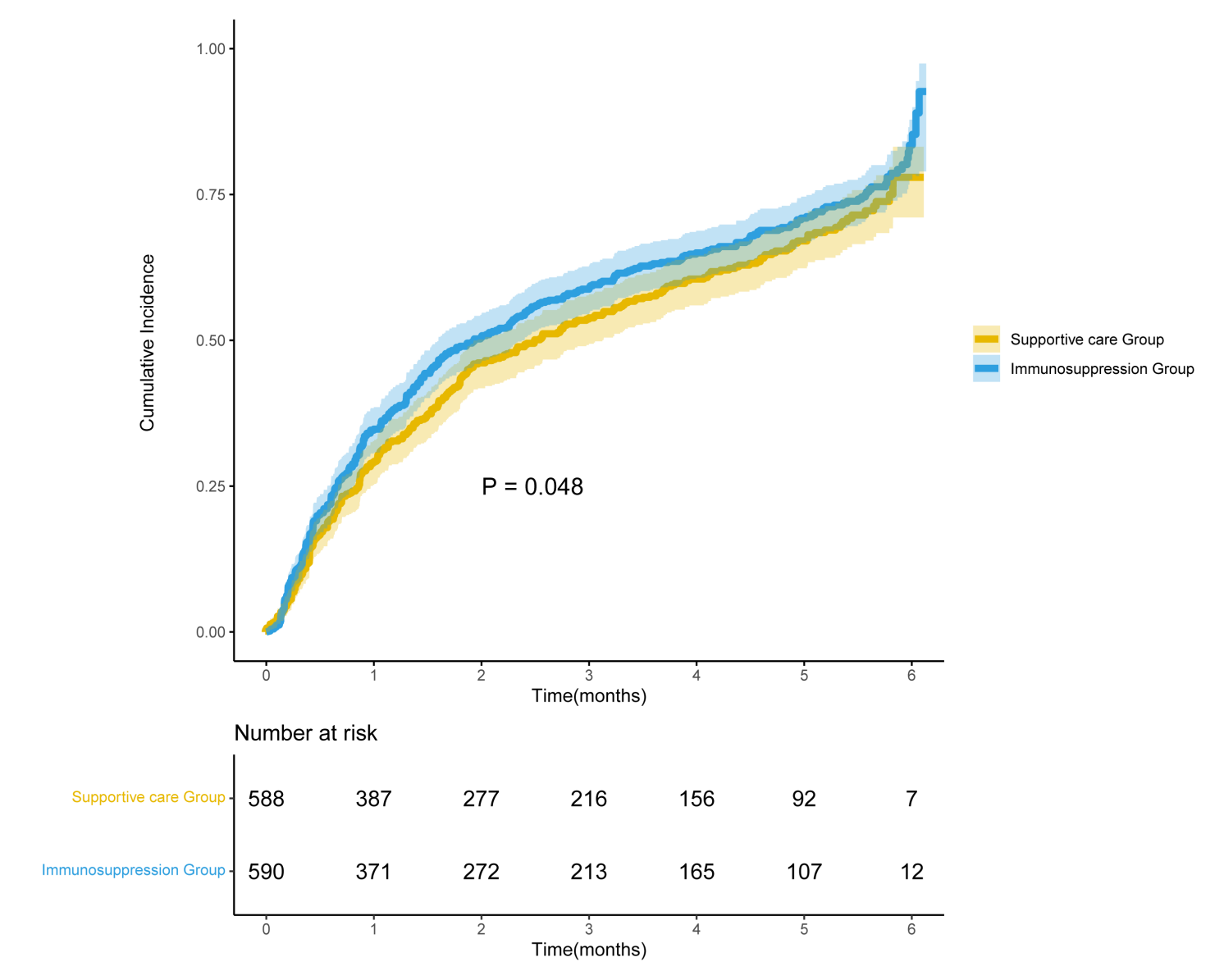


Alt Text: The proteinuria was reduced among patients who received immunotherapy .

**Supplementary Figure 2. The estimated effects of immunosuppressants on the secondary outcome in subgroups Hazard ratio(adjusted for age, sex, eGFR, proteinuria, SumMEC score, S score, and T score).**


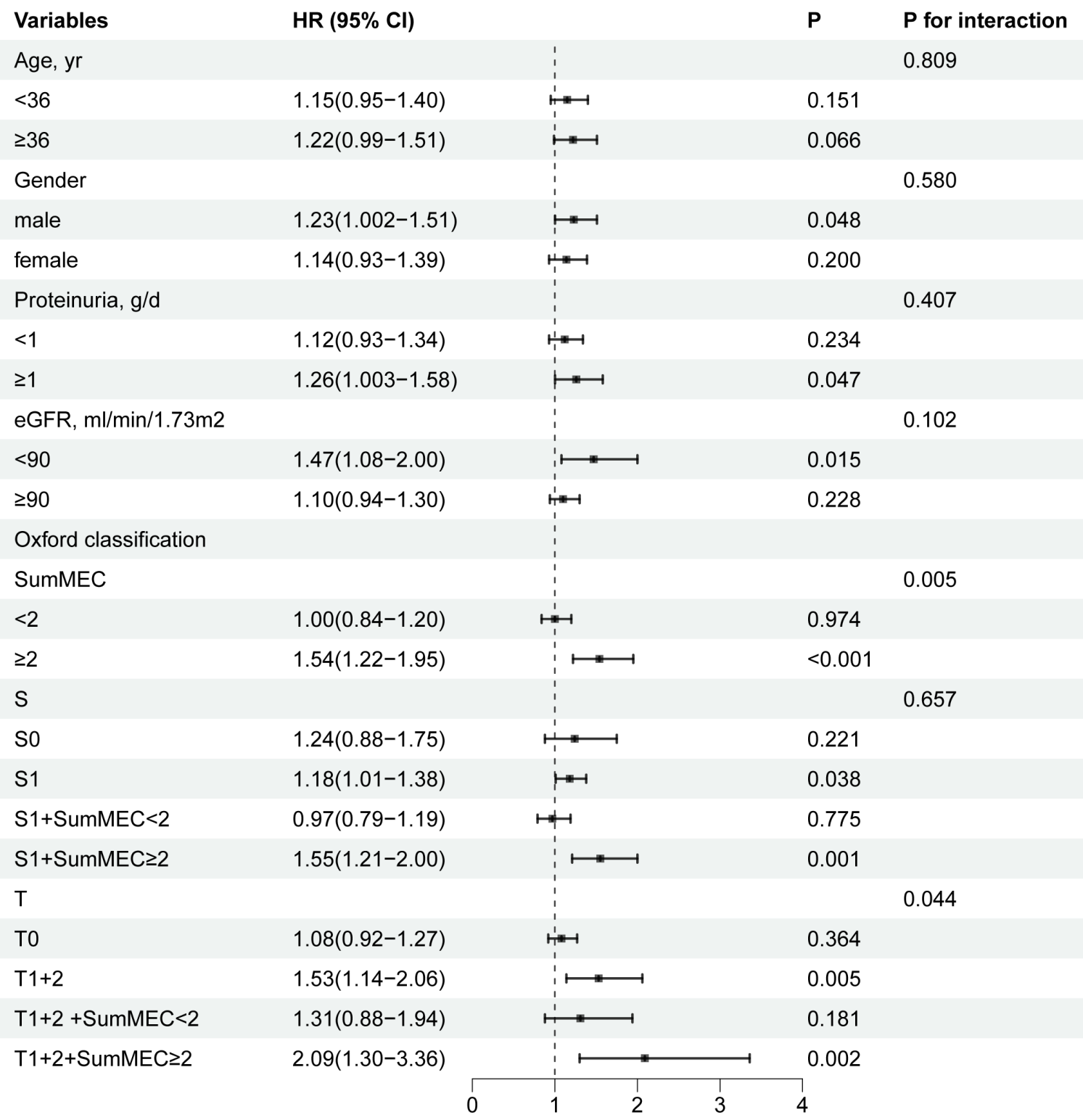


Alt Text: Immunotherapy improved proteinuria remission among patients in the SumMEC ≥ 2 subgroup, as well as the patients in S1 or T1+2 subgroup when these patients existed a concomitant SumMEC score ≥ 2.
